# Supplementary material for: Functional coupling between TRPV4 channel and TMEM16F modulates human trophoblast fusion
Source: eLife. 2022 Jun 7;11:e78840. doi: 10.7554/eLife.78840 (PMC9236608; doi:10.7554/eLife.78840)
Supplement: Supplementary file 1. [file elife-78840-supp1.docx]

**Supplementary File 1. qPCR primer list used in this study.**

| **Target** | **Forward, 5’→3’** | **Reverse, 5’→3’** |
| --- | --- | --- |
| Actin | ACTGGGACGACATGGAGAAAAA | GCCACACGCAGCTC |
| CGB | CTTCCAGGACTCCTCTTC | TTTTTGCGGATTGAGAAGC |
| CSH1 | CTATCACCTCCTAAAGGACC | TTGTGTCAAACTTGCTGTAG |
| Syncytin-1 | ATGGAGCCCAAGATGCAG | AGATCGTGGGCTAGCAG |
| TMEM16F | AAATTGCCTCTGAAACCCAATGA | GCTTTCGTCTACACTGAGGACTT |
| TRPV4 | GATGGGCGACCAAATCTGC | GAGGACTCATATAGGGTGGACTC |
| GAPDH | CATGAGAAGTATGACAACAGCCT | AGTCCTTCCACGATACCAAAGT |
